# Is Oxytocin a Contributor to Behavioral and Metabolic Features in Prader–Willi Syndrome?

**DOI:** 10.3390/cimb46080518

**Published:** 2024-08-13

**Authors:** Maria Petersson, Charlotte Höybye

**Affiliations:** 1 Department of Endocrinology, Karolinska University Hospital, 171 76 Stockholm, Sweden; charlotte.hoybye@ki.se; 2 Department of Molecular Medicine and Surgery, Karolinska Institutet, 171 76 Stockholm, Sweden

**Keywords:** PWS, oxytocin, metabolism, weight, behavior, social interaction, hyperphagia

## Abstract

Prader–Willi Syndrome (PWS) is a rare genetic disorder typically characterized by decreased social interaction, hyperphagia, poor behavioral control and temper tantrums, together with a high risk of morbid obesity unless food intake is controlled. The genetic defects that cause PWS include paternal 15q deletion (estimated in 60% of cases), chromosome 15 maternal uniparental disomy (UPD) (estimated in 35% of cases) and imprinting defects and translocations. Several studies indicate an oxytocin deficiency in PWS. Oxytocin is a hypothalamic nonapeptide with receptors located in the brain and in various other tissues in the body. It acts as a neuropeptide in several brain areas of great importance for behavioral and metabolic effects, as well as a neurohypophyseal hormone released into the circulation. Oxytocin in both rats and humans has strong and long-lasting behavioral and metabolic effects. Thus, an oxytocin deficiency might be involved in several of the behavioral and metabolic symptoms characterizing PWS. Treatment with oxytocin has, in some studies, shown improvement in psycho-social behavior and hyperphagia in individuals with PWS. This review focus on the behavioral and metabolic effects of oxytocin, the symptoms of a potential oxytocin deficiency in PWS and the effects of oxytocin treatment.

## 1. Introduction

Prader–Willi syndrome (PWS) is a rare, genetic, neurodevelopmental disorder with a complex phenotype. It occurs in approximately 1 in 10,000 to 1 in 30,000 births and is caused by a defect in the paternal expression of imprinted genes in chromosome 15 in the region q11–13. The most common defect (60%) is paternal deletion, followed by maternal disomy (35%). The remaining defects are imprinting defects, translocations, or inversions. The majority of cases occur spontaneously, and the incidence is anticipated to be the same worldwide, although data are only available for a few countries. PWS is characterized by muscular hypotonia, motor, and cognitive developmental delays, mild to moderate intellectual disability, behavioral problems, including anxiety, compulsive and controlling behavior and temper outbursts. Hormone deficiencies are common, in particular, hypogonadism and growth hormone (GH) deficiency, leading to a short stature unless GH is administered during childhood. Most infants with PWS have feeding problems, which are switched to hyperphagia at the age of 4 to 6 years. Body composition is abnormal with more body fat than muscle mass, and the metabolic rate is low. Due to the hyperphagia, a strict diet and supervised food intake are mandatory to prevent extreme obesity and related comorbidity [1].

PWS is believed to be a hypothalamic disease, and studies of postmortem brain tissue from adults with PWS have shown a 42% decrease in number and a 54% decrease in volume of oxytocin-expressing neurons in the paraventricular nucleus (PVN) of the hypothalamus suggested to be the cause for the insatiable hunger and decreased satiety in PWS [2,3]. Very low concentrations of oxytocin in lymphoblastoid cells and in postmortem frontal cortex tissue were also demonstrated [4]. Thus, low plasma levels of oxytocin would be expected, but oxytocin is often reported to be increased in children with PWS. In a study of plasma levels of oxytocin, 23 children with PWS were compared with 18 healthy unrelated siblings matched for age and with a similar gender ratio and BMI. The children with PWS were found to have more than twice as high levels of plasma oxytocin as compared to unrelated siblings [5], and analyses of serum oxytocin in adults with PWS showed similar concentrations as in controls, but, in relation to their obesity, the concentrations were low [6]. In addition, analyses of oxytocin in cerebrospinal fluid showed higher concentrations in adolescents and adults with PWS compared to the controls [7].

The 15q11–13 region contains both protein-coding DNA genes and non-coding RNA genes. Mutations or deletions of genes in this region (Magel2 and Necdin) are in mouse models associated with hypotonia, developmental delay, hypogonadism, hyperphagia and impaired social skills. A pronounced dysregulation of the signaling pathways for oxytocin has been shown in a mouse model of PWS, and, in this model, an early postnatal administration of oxytocin improved social behavior [8].

In humans with PWS, several trials studying the intranasal administration of oxytocin have been performed. The study cohorts varied regarding age, oxytocin dose and gender, and the studies have shown varying degrees of efficacy on hyperphagia, anxiousness, distress and social behavior. In a recent systematic review and meta-analysis on the efficacy of intranasal oxytocin in individuals with PWS, four studies with 56 individuals receiving oxytocin, and 61 individuals receiving placebo, were included [9]. Pooled data from 92 individuals did not show a better effect of oxytocin on hyperphagia compared to placebo. In the same meta-analysis, pooled data from 94 individuals showed that the effect of oxytocin on weight was not different from placebo. However, in studies of children <11 years, oxytocin significantly reduced hyperphagia, especially in boys, whereas no effect was observed in individuals between 13 and 29 years suggesting a better effect in younger children [9].

Oxytocin is a neurotransmitter and neurohypophyseal hormone. Besides the classical effects on uterine contraction and milk ejection during labor and breastfeeding, oxytocin has many other effects. Oxytocin has behavioral and metabolic effects in both sexes and has been suggested to influence, for example, anxiety and compulsive and social behavior, as well as eating behavior. It also interacts with a number of other neuropeptides and hormones of importance for behavior and metabolism. Thus, a decrease or change in oxytocin synthesis or distribution could be involved in several of the symptoms in PWS described above. In the present review, similarities between low levels of oxytocin and symptoms in PWS and their possible relationships will be discussed.

## 2. Clinical Features and Nutritional Phases in PWS

PWS is a multisymptomatic syndrome with clinical features varying with age (Table 1) [10,11]. Typical dysmorphic features include a long narrow face, narrow bifrontal diameter, almond-shaped palpebral fissures, and a small mouth with a thin upper lip, small feet for height and age and small narrow hands with a straight ulnar border. The neonatal period is characterized by profound muscular hypotonia, a poor sucking reflex and failure to thrive. During infancy, muscular hypotonia improves, and a mild to moderate intellectual dysfunction and delayed psychomotor development become increasingly apparent along with impaired skeletal growth. Central and obstructive sleep apnea as well as excessive daytime sleepiness are common. During childhood, hyperphagia develops and a constantly supervised and restricted diet together with scheduled daily physical activities are necessary to avoid morbid obesity. Body composition is abnormal in PWS with more body fat than lean body mass. Most patients with PWS have genital hypoplasia and hypogonadism, which can be of both primary gonadal and hypothalamic origin. Delayed and incomplete pubertal development is common, and a decreased or absent growth spurt results in reduced final adult height. Typically starting in childhood are temper tantrums, a stubborn and controlling behavior, compulsive–obsessive features and a very strong preference for routines. Psychiatric and behavioral problems become more prominent with increasing age and are, together with the patient’s hyperphagia and cognitive disabilities, the main limitations to independent living for adults with PWS.

PWS is characterized by severe hypotonia and failure to thrive in infancy, which is replaced by progressive hyperphagia and a high risk of development of morbid obesity. For many years, only those two nutritional phases were believed to be present in PWS. However, in 2011, Miller et al. described seven different nutritional phases, five main phases and two sub-phases [12]. This description of the nutritional phases in PWS has greatly improved our understanding of its natural history. Briefly, phase 0 occurs in utero, with decreased fetal movements and growth restriction. In phase 1, the infant is hypotonic and not obese. In sub-phase 1a, the infant has difficulty eating with or without failure to thrive (birth to 15 months). In sub-phase 1b, the infant grows steadily, and weight is increasing at a normal rate (the median age of onset: 9 months). In phase 2, weight increases. In sub-phase 2a, the weight increases without a significant change in appetite or caloric intake (the median age of onset: 2 years), while, in sub-phase 2b, the weight gain is associated with an increased interest in food (the median age of onset: 4.5 years). Phase 3 is characterized by hyperphagia, with the typical food-seeking behavior and a lack of satiety (the median age of onset: 8 years). Some adults progress to phase 4 where the appetite is no longer insatiable. PWS is a complex model to study and understand the development of obesity and appetite regulation due to the different nutritional phases that individuals with PWS undergo, from anorexia and failure to thrive in phase 1a to hyperphagia and the lack of satiety in phase 3.

## 3. Oxytocin

Oxytocin is a nonapeptide produced within two hypothalamic nuclei, the supraoptical nucleus (SON) and the PVN. There is a broad spectrum of nerves connecting the neurons within the PVN with neurons in several brain areas including other parts of the hypothalamus such as the ventromedial nucleus and the arcuate nucleus and the brainstem where also oxytocin receptors are located. Oxytocin receptors are also found in many other areas within the body for example the endocrine pancreas, the adipose tissue, liver, muscles as well as the gastrointestinal tract [13,14,15,16,17].

In addition to being released during parturition and breastfeeding oxytocin is released in response to a number of other stimuli, induces a multitude of effects and influences several other hormones and neurotransmitters. Besides being released as a hormone from the neurohypophysis, oxytocin is as mentioned above, released into the brain and then not only from the axon into the synaptic cleft but also from the dendrites spreading oxytocin in the extracellular fluid making it possible to reach larger areas. It has even been shown that oxytocin may be produced within the dendrites [18].

Oxytocin is measurable in human plasma but of importance to remember is that concentrations in peripheral blood do not always reflect the concentrations within the CNS or the cerebrospinal fluid.

In this review we will focus on the effects of oxytocin on metabolism and behavior. Such effects were demonstrated during the 1960s–1980s, when oxytocin was reported to induce maternal and sexual behavior, to influence feeding and foraging, gluconeogenesis and glucogenolysis, as well as the release of insulin and several gastrointestinal hormones (for a review see for example [19,20].

## 4. Oxytocin and Metabolism

Oxytocin may, during certain circumstances, induce insulin-like effects where it stimulates glucose uptake, as well as lipogenesis, through effects on oxytocin receptors in the adipose tissue [13,21,22]. On the other hand, oxytocin has also been demonstrated to be released in response to hypoglycemia and to increase blood glucose [21,23] by stimulating both glycogenolysis and gluconeogenesis [24,25], and it may also stimulate lipolysis [26]. Interestingly, the effect on lipolysis has been suggested to be induced by a subpopulation of oxytocinergic neurons located in the adipose tissue interacting with noradrenaline release [26].

Oxytocin has also been demonstrated to release insulin through effects within the endocrine pancreas [27] and the dorsal motor nucleus (DMX) [28]. However, an opposite effect on insulin release has been demonstrated when oxytocin is injected into the dorsal vagal complex (DVC) where oxytocin decreases insulin [29]. In rats, the increase in glucose and glucagon during suckling was attenuated in response to an oxytocin antagonist [28]. It is reasonable that oxytocin may have dual effects on metabolism: one during breastfeeding and another during nonpregnant/nonbreastfeeding states, and it has been suggested that oxytocin may take part in the mobilization and transfer of energy in the production of milk during lactation [30]. Oxytocin increases the proliferation and differentiation of osteoblasts [31,32], and, in muscles, oxytocin seems to have both anabolic and protective effects [33]. An anabolic effect of oxytocin on bone and muscles during breastfeeding might serve to protect the breastfeeding mother against a too large breakdown of bone and muscles during this period with high prolactin and decreased estrogen levels. In a short-term perspective, both the stimulating and inhibiting effects of oxytocin on the release of GH and insulin-like growth factor-1 (IGF-1) have been demonstrated, but, in a long-term perspective, oxytocin seems to increase the plasma levels of IGF-1 [34,35].

Similarly, opposite effects of oxytocin have been reported on food intake and weight gain. In female rats, these effects are dependent on estrogen levels, dose and the route of administration [36,37].

In ovariectomized (OVX) rats, a decrease in weight in response to oxytocin has been demonstrated [38,39]. In humans, lower levels of oxytocin were found in postmenopausal women compared to premenopausal women and in obese women compared to women of normal weight [40]. Several recent studies have focused on oxytocin as a weight reducing hormone, changing metabolism, decreasing food intake and increasing energy expenditure [41]. However, from a physiological point of view this would not be advantageous during pregnancy and breastfeeding, but, as discussed above, oxytocin might have different effects depending on the situation and hormonal background.

Oxytocin receptors and oxytocinergic neurons projecting to other areas within the hypothalamus and the brainstem have been demonstrated, and, within these areas, oxytocin can influence metabolism through effects on other hormones and neurotransmitters, as well as the activity within the autonomic nervous system. Within the hypothalamus, oxytocinergic neurons reach other nuclei of importance for metabolism such as the ventromedial hypothalamic nucleus and the arcuate nuclei, and, in the brainstem, both vagal and sympathetic areas are reached by the oxytocinergic neurons where oxytocin receptors are also located. Within the hypothalamus, oxytocin has also been demonstrated to be released from somatodendrites spreading oxytocin within this area [14,16,42]. Oxytocin receptors are widespread in the periphery and present in, for example, the liver and the gastrointestinal tract, the endocrine part of the pancreas and muscles, as well as adipocytes [14,15,16,17]. The increase in metabolism and decrease in food intake have been suggested to be induced by an increase in activity within the sympathetic nervous system [41]. Indeed, in a mouse model deficient in oxytocin receptors, a low sympathetic tone and increased weight without an increase in food intake were seen [43]. An increase in activity within the sympathetic nervous system related to oxytocin might be true during certain circumstances, although, in contrast, oxytocin is also referred to as an anti-stress hormone since it decreases blood pressure and the activity within the hypothalamic–pituitary–adrenal (HPA)-axis. Additionally, during breastfeeding, oxytocin increases vagal nerve activity. Moreover, in rats, oxytocin decreases the activity within the locus coeruleus and increases the responsiveness of alpha 2-adrenoreceptors [44,45,46]. When oxytocin is administered postnatally to rats, a lifelong change with lower blood pressure and increased responsiveness of alpha 2-adrenoreceptors have been reported, while changes in weight seem to depend on sex and strain [47,48,49].

In hypothalamus, both the melanocyte stimulating hormone (MSH) and leptin have been demonstrated to affect oxytocin release from the PVN [50,51]. There seems also to be correlations and interactions between GLP-1 and oxytocin [52,53]. When oxytocin was administered to rats, an increase in the hunger hormone ghrelin [54] and lower levels of thyroid hormones have been reported [55]. In contrast, individuals with PWS have higher ghrelin levels and, more frequently, a central hypothyreosis compared to non-PWS individuals. Concerning the effects of oxytocin on body weight, conflicting results have been described in an overview of studies of oxytocin in humans [41]. Interestingly, in a study on obese non-PWS individuals treated with oxytocin intranasally four times a day for 8 weeks, weight decreased significantly, with the most pronounced weight loss in the most obese individuals. In individuals with a normal BMI, no weight loss was seen [56]. Furthermore, in diabetic mice, oxytocin administration decreased weight and food intake and improved glucose and fat metabolism. Meanwhile, his was not observed in non-diabetic controls [57].

Thus, as discussed above, oxytocin might have different metabolic effects depending on the physiological and experimental context. Effects may differ, for example, depending on time, gender, age, other hormones or stress, as well as if the individual is in a fed or fasting state.

In PWS, metabolism is decreased, and body composition is different, with more body fat than lean body mass, while glucose metabolism is usually normal [10]. It is likely that oxytocin, through various central and peripheral effects, plays a role for at least some of the symptoms seen in PWS, although there are several other hormones that are stronger regulators of metabolism.

## 5. Oxytocin and Behavior

The first behavioral effects of oxytocin that were described were the effects on maternal behavior and bonding between mother and offspring/child [58,59]. Oxytocin is released not only in response to the milk ejection reflex but also in response to the early skin-to-skin contact between mother and child, which are important for the bonding. A similar release of oxytocin in response to skin-to-skin contact has been demonstrated also in fathers [60,61].

As discussed above, in relation to the effects of oxytocin on metabolism and weight, likewise, oxytocin seems to have dual effects also in some aspects of behavior. In rats, oxytocin can induce both anxiolytic-like and sedative effects depending on dose and the route of administration but also depending on the stage of the estrous cycle [37,62], and oxytocin administered postnatally to pigs and rats induces lifelong behavioral changes [47,49,63].

In rats and mice, oxytocin induces grooming, sexual and social behavior, and it decreases aggression. Animal studies suggest that oxytocin increases social interaction by decreasing activity within the amygdala. In an animal model of depression, oxytocin has been found to induce similar effects as antidepressant medication [64].

There are many studies indicating that oxytocin plays a role for several behaviors in humans since oxytocin levels have been demonstrated to be associated with social behavior, anxiety, sexual activity, depression, trust and decision making (for reviews, see, for example [19,65]).

Oxytocin levels are lower in female patients with depression [66], and, in some but not all studies, oxytocin has been reported to improve depression [67,68,69].

In 2005, Kosfeld et al. showed that oxytocin administered intranasally could increase trust in humans, and, approximately ten years later, Lambert al. used an MRI to demonstrate that oxytocin affects decision making [70,71].

In another experimental setting, children exposed to a situation of social stress were tested. As a result, the children who could see or hear their mother’s voice had higher levels of oxytocin and lower levels of cortisol compared to the children who had no contact with their mother [72].

Oxytocin has also been suggested to play a role in the symptoms related to autism spectrum disorders [73], and, interestingly, oxytocin was found to reduce increased activity in the amygdala in response to angry faces in participants with autism-spectrum disorders [74].

In a meta-analysis [75], lower oxytocin levels were found in children but not in adolescents with autism, and, in some studies, the administration of oxytocin was reported to improve social behavior in these individuals. In a mouse model of autism, oxytocin administered neonatally restored social behavior [76].

The above-mentioned effects might be induced by oxytocin itself, but it could also involve other hormones and neurotransmitters. Oxytocin neurons reach several brain areas of importance for behavior such as the frontal cortex, the amygdala, the hippocampus and the bed nucleus of the stria terminalis where oxytocin receptors are expressed [14,16].

For example, oxytocin increases the activity within 5HT1a/5HT2a and 5HT2c-receptors [77,78,79], as well as the responsiveness of alpha 2-adrenoreceptors, and decreases the activity within the HPA axis. In rats, oxytocin can affect the number of glucocorticoid receptors within for example the hippocampus [80].

In addition, oxytocin may influence dopaminergic receptors and dopaminergic transmission [81], and the effect on anxiety by oxytocin has been suggested to involve GABA-A-receptors [82].

It could be speculated that a deficiency or change in oxytocin levels or signaling might explain some of the behavioral challenges seen in PWS. Low levels of oxytocin are associated with anxiety, less social interaction, and less calmness, all behaviors common in PWS. In addition, individuals with PWS have changes in their GABA-A-receptors, a lower activity within the HPA axis and the serotoninergic transmission where especially the 5HT2C-receptors have been thought to play a role in PWS [83]. Similar changes have also been linked to oxytocin levels or oxytocin administration as discussed above.

In accordance with the effect of oxytocin administered early in life that were discussed above, oxytocin administered within 5 min after birth in a mouse model of PWS induced long-term changes since it normalized both learning and memory as well as social cognition in the mice [84]. Altogether, the behavioral alterations in PWS and the effects of oxytocin observed on those have been a great inspiration for clinical trials of oxytocin in PWS, as described below.

## 6. Clinical Trials in PWS

### 6.1. Clinical Trials on the Use of Oxytocin in PWS

The first clinical trial of oxytocin in PWS was performed by Tauber et al. in 2011. Twenty-four individuals, with a median age of 28.5 years, were included. Every individual received a single dose (24 IU) of either oxytocin or a placebo. A questionnaire was developed to assess ten social and emotional behaviors, with four questions to assess eating behavior. A first assessment of the individual’s behavior was performed two days before drug administration, a second at 12 h after administration for evaluation of early effects and a third at two days after drug administration for an evaluation of late effects. There was no difference between the two groups before drug administration, but a significant increase in trust and tendencies to be less sad and conduct disruptive behavior was seen two days after oxytocin administration. No statistical difference was observed in the eating behavior scores between the groups, but there was improvement in social skills in the oxytocin-treated group [85].

In 2014, Einfeld et al. reported on a study of 30 individuals with PWS included in an 18-week double-blind, randomized, placebo-controlled trial with an intranasal use of oxytocin to examine its effect on physical, behavioral, and cognitive function. For the assessments, the Developmental Behavior Checklist (DBC), the Yale–Brown Obsessive Compulsive Scale (Y-BOCS), the Dykens Hyperphagia Questionnaire, the Reading the Mind in the Eyes Test (RMET) and the Epworth Sleepiness Scale (ESS) were used. Two different doses of oxytocin were administered for 8 weeks according to the age of the individuals. Individuals aged 16 years and above (*n* = 11) received 24 IU twice daily, and individuals between 13 and 15 years received 18 IU twice daily. This period was followed by a two-week washout period. For the following eight weeks, the dose was increased to 40 IU twice daily for individuals older than 16 years (*n* = 18) and 32 IU twice daily for individuals between 13 and 15 years. The study did not show improvement in behavioral problems or in hyperphagia in response to the oxytocin treatment. Unexpectedly, an increase in temper outbursts was noted with higher doses of oxytocin [86].

In 2016, in a randomized, double-blind, placebo-controlled, cross-over study conducted on 25 children with PWS was performed by Kuppens et al. The aim was to examine the effect of oxytocin on eating and social behavior. For the assessments, the Dykens Hyperphagia Questionnaire and the Oxytocin Study Questionnaire were used. Depending on the age, the participants received either oxytocin 24–48 IU or a placebo divided in two daily doses for four weeks. They then crossed over to the opposite treatment for another four weeks. The analyses of children <11 years compared to children >11 years did not show any differences between treatment with oxytocin or placebo. However, in children <11 years, oxytocin compared to a placebo had beneficial effects on hyperphagia and social behavior, and the children showed less anger and sadness [87].

In 2017, Miller et al. conducted a double-blind, placebo-controlled, cross-over trial in 24 children, 5 to 12 years old, with PWS. The participants received 16 IU of intranasal oxytocin or a placebo for five days, followed by a four-week washout period, and then they switched to the opposite treatment for another five days. The Aberrant Behavior Checklist, Social Responsiveness Scale (SRS-P), Repetitive Behavior Scale—Revised (RBS-R), Hyperphagia Questionnaire, and the Clinical Global Impression (CGI) scale were used to evaluate hyperphagia and other behaviors. The authors concluded that a five-day treatment with a low dose of oxytocin in children was safe, and, compared to the placebo treatment, improvements were noted in anxiety and self-injurious behaviors [88].

In 2017, Tauber et al. reported results from a single dose of intranasal oxytocin administered to infants less than six months of age. Sucking and swallowing were examined before and after the oxytocin administration by using the Neonatal Oral Motor Assessment Scale (NOMAS), video fluoroscopy of swallowing, and the Clinical Global Impression (CGI) scale. Oxytocin was well tolerated, with no side effects noted during the seven days of treatment, and oxytocin improved oral feeding and social skills [89].

In 2021, Damen et al. performed a randomized, double-blind, placebo-controlled, cross-over study on the effects of oxytocin versus the placebo treatment for three months in children with PWS to investigate changes in social behaviors and hyperphagia, with differences between males and females and between PWS molecular genetic classes. The social and eating behaviors were evaluated with the Oxytocin Study Questionnaire, the Dykens Hyperphagia Questionnaire and the RBS-R. Forty-six children with PWS, 3 to 11 years old, were randomized to intranasal oxytocin 16–40 IU or a placebo daily divided in two doses for three months. No significant effects on hyperphagia or social behavior were seen. However, subanalyses showed a more pronounced effect of oxytocin on hyperphagia in individuals with 15q11-q13 deletions and in boys [90].

In another study the same year, Hollander et al. studied the effect of intranasal oxytocin on hyperphagia and repetitive behaviors in 23 children with PWS, 5 to 18 years of age. The hyperphagia and repetitive behaviors were evaluated with the Dykens Hyperphagia Questionnaire form and the RBS-R. The trial was randomized, double-blind, placebo-controlled and lasted eight weeks. The participants were assigned to 16 IU oxytocin or a placebo per day. Modest improvements in hyperphagia and repetitive behaviors were observed in the placebo group, while no changes were seen in the oxytocin group [91].

### 6.2. Clinical Trials on the Use of Carbetocin in PWS

Studies with carbetocin, an oxytocin analog with greater selectivity for oxytocin receptors and a longer half-life, have been performed and are ongoing. Carbetocin is a well-known substance, approved since 1997, to prevent uterine atony following caesarean delivery. In a 14-day phase 2 trial that was randomized, double-blind and placebo-controlled, 37 individuals with PWS, 10 to 18 years old, were assigned to carbetocin (*n* = 17) or a placebo (*n* = 29). The participants received 9.6 mg of intranasal carbetocin three times per day or a placebo. The study showed a decrease in hyperphagia and other behavioral symptoms in response to carbetocin, and it was safe and well tolerated [92]).

In a recent phase 3 trial, a total of 130 participants with PWS aged 7 to 18 years were enrolled. The participants were randomized to 9.6 mg/dose carbetocin (*n* = 44), 3.2 mg/dose carbetocin (*n* = 43) or a placebo (*n* = 43) three times daily during 8 weeks. During a subsequent 56-week long-term follow-up period, the placebo participants were randomly assigned to 9.6 mg (*n* = 64) or 3.2 mg carbetocin (*n* = 64), while carbetocin participants continued at their previous dose. Due to the COVID-19 pandemic, enrolment was discontinued prematurely, but the study showed that carbetocin was well tolerated and that the 3.2 mg dose was associated with clinically meaningful improvements in hyperphagia as well as anxiousness and distress behaviors [93].

In summary, beneficial effects of oxytocin treatment in PWS were seen in seven out of nine studies (Table 2). The positive effects consisted of improvements in hyperphagia and distress behaviors. However, the results of the studies cannot be compared straight away as the study designs varied, the study cohorts consisted of different age groups with different gender compositions, different doses and numbers of administrations of oxytocin were used, the duration of the studies varied and, in some studies, a limited number of participants were included. In addition, the long-term effects and risk of adverse effects in humans are not known. From animal experiments, we know that oxytocin can induce long-lasting, perhaps lifelong, behavioral and metabolic effects. The knowledge of long-term consequences are of major importance, in particular, in PWS patients due to intellectual disability, behavioral challenges and often problems describing their health and health care needs.

## 7. Conclusions

Oxytocin has a lot of effects on metabolism and behavior, including hyperphagia, but the magnitude of these effects depends on the situation and context. These symptoms are a major challenge in PWS, and, as studies have demonstrated low concentrations of oxytocin in PWS, oxytocin is, therefore, potentially an attractive treatment. In some of the clinical trials performed with oxytocin on individuals with PWS, anxiety and compulsiveness decreased. Hyperphagia could be considered as a compulsive behavior, and it also improved in some of the studies. Recent studies with the oxytocin analogue carbetocin showed similar effects with improvements in hyperphagia and behavior. Worth mentioning is that, in non-PWS individuals with obsessive compulsive disorder (OCD), changes in the oxytocin receptor have been found, and a methylation of the oxytocin receptor has even been suggested as a biomarker for the response to the treatment of OCD [94].

To our knowledge, no study has shown an effect of oxytocin on weight in PWS. However, if oxytocin is administered during a longer period, it might, at least in younger patients, have the potential to decrease weight or protect against further weight gain since oxytocin, as discussed above, in several studies decreased hyperphagia. Thus, oxytocin seems to positively affect behavior in PWS, at least during certain circumstances and especially if administered early in life.

## 8. Future Directions

Oxytocin is an interesting peptide that seems to modulate and balance many behavioral and metabolic effects to keep the homeostasis. Since oxytocin is influenced by and is influencing several other neurotransmitters and hormones, the effects seen in response to oxytocin may not always be caused by oxytocin itself. Oxytocin interacts directly or indirectly with GABA-receptors, 5HT-receptors, dopamine receptors, glucocorticoid receptors and the HPA axis, as well as adrenergic receptors and the autonomic nervous system. To further understand the role of oxytocin in PWS, more studies of these interactions are needed as well as studies of the effects of oxytocin during different ages and in the PWS of different genotype. For example, PWS individuals with maternal disomy 15 often have delayed diagnosis, higher verbal IQ scores with greater attention, factual knowledge and better social reasoning skills than those with the typical Type I or Type II deletions involving the 15q11-q13 region. On the other hand, patients with maternal disomy are more prone to increased episodes of psychosis and autistic behaviors [10]. Indeed, in a study by Damen et al. [90], an effect of oxytocin was only seen in the PWS with the 15q11-q13 deletion and not in the PWS with the maternal disomy, but these results need to be repeated in larger cohorts. Further studies of different ages are also of importance since oxytocin seems to have effects in particular in the youngest children. Perhaps it should be administered as early as during the neonatal period since oxytocin, when administered early in life, in animal experiments induces long-lasting and even lifelong effects, but more studies are needed. Also, the beneficial effects of oxytocin in human infants younger than 6 months need to be better established.

Other unanswered questions are what dose of oxytocin to use to be the most effective and what the long-term consequences of oxytocin treatment are. Oxytocin has a very short half-life. Different doses and administration intervals have been used in previous studies. Carbetocin has an increased half-life compared to oxytocin and crosses the blood–brain barrier more easily. It is unclear if diurnal fluctuations in oxytocin concentrations are an asset and if high concentrations can introduce a state of resistance. Future studies with varying doses and numbers of administrations will be of value to answer these questions.

## Figures and Tables

**Table 1:** Most frequent characteristics of PWS.

Behavioral challenges	Feeding difficulties and failure to thrive in infancy
	Hyperphagia
	Poor social skills
	Temper outbursts
	Anxiety
	Compulsive behaviors
Cognition/intellectual capacity	Mild to moderate intellectual and cognitive dysfunction
Physical features	Profound hypotonia in infancy
	Short stature
	Genital hypoplasia
	Obesity
Endocrine changes	Growth hormone deficiency
	Hypogonadism

**Table 2:** Summary of clinical trials of oxytocin and carbetocin in PWS.

Dose/Duration and Study Design	Number and Gender	Age	Effect	Authors
24 IU single dose	*N* = 24 (16 females)	18–43 years	Trust increase	Tauber et al., 2011 [85]
18–32 IU twice daily and 24–40IU twice daily for 8 weeks Randomized, double-blind, placebo-controlled and open-label extension	*N* = 29 (9 females)	13–15 years and 16–29 years	No effect (increase in temper outbursts with high dose)	Einfeld et al., 2014 [86]
24–48 IU for 4 weeks Randomized, double-blind, crossover	*N* = 25 (11 females)	6–14 years	Less hyperphagia, social behavior improved	Kuppens et al., 2016 [87]
16 IU for 5 days Randomized, double-blind, crossover	*N* = 24 (12 females)	5–12 years	Less anxiety and self-injury	Miller et al., 2017 [88]
7 IU for 7 days Case cohort study	*N* = 18 (8 females)	<6 months	Improved oral feeding and social skills	Tauber et al., 2017 [89]
16–40 IU for 12 weeks Randomized, double-blind, crossover	*N* = 26 (13 females)	3–11 years	Less hyperphagia in 15q-11–13 and boys	Damen et al., 2021 [90]
16 IU for 8 weeks Randomized, double-blind, placeboc-ontrolled	*N* = 23 (5 females)	5–18 years	No effect	Hollander et al., 2021 [91]
Carbetocin 9.6 mg three times daily for 2 weeks Randomized, double-blind, placebo-controlled	*N* = 37 (23 females)	10–18 years	Less hyperphagia	Dykens et al., 2021 [92]
Carbetocin 9.6 mg or 3.2 mg three times daily for 8 and 56 weeks Randomized, double-blind, placebo-controlled and open-label extension	*N* = 130 (66 females)	7–18 years	Low dose: less hyperphagia and anxiety	Roof et al., 2023 [93]
